# Urolithin A Exerts Neuroprotective Effects Against Ischemic Stroke by Inhibiting Oxidative Stress and Neuroinflammation

**DOI:** 10.1002/cns.71081

**Published:** 2026-08-12

**Authors:** Yanxin Shao, Lina Feng, Yanchun Li, Yichen Cai, Hui Yuan, Qin Tang, Mingfeng Yang, Weixia Yang, Hong Shi, Leilei Mao

**Affiliations:** ^1^ The Second Affiliated Hospital Shandong First Medical University & Shandong Academy of Medical Sciences Tai'an Shandong Province China; ^2^ Department of Anesthesiology, Shanghai Pulmonary Hospital, School of Medicine Tongji University Shanghai China; ^3^ Department of Neurology The Third Affiliated Hospital of Qiqihar Medical University Qiqihar Heilongjiang China; ^4^ Department of Neurology Qingpu Branch of Zhongshan Hospital Affiliated to Fudan University Shanghai China; ^5^ The National Clinical Research Center for Aging and Medicine, Department of Hand and Upper Extremity Surgery, Jing'an District Central Hospital Fudan University Shanghai China

**Keywords:** HO‐1, ischemic stroke, neuroinflammation, Nrf2, oxidative stress, Urolithin A

## Abstract

**Background:**

Ischemic stroke (IS), the main stroke type, causes neuronal injury via hypoperfusion and hypoxia, imposes heavy health burdens, and is characterized by neurological deficits driven by oxidative stress and inflammation.

**Methods:**

A rat model of IS was established via middle cerebral artery occlusion (MCAO). Sensorimotor and cognitive dysfunctions post‐IS were assessed using neurological function scoring, the rotarod test, the adhesive removal test, the foot‐fault test, and the Morris water maze (MWM) test. Brain injury was evaluated by TTC staining, immunofluorescence staining, and Western blotting. For mechanistic exploration, RNA transcriptome sequencing analysis, immunofluorescence staining, and ELISA were employed to determine UA's effects on oxidative stress and neuroinflammation following IS.

**Results:**

UA treatment was confirmed to exert neuroprotective effects against ischemic stroke, as it can inhibit neuronal injury and improve sensorimotor and cognitive functions in rats. Mechanistically, UA upregulates the expression of Nrf2/HO‐1, thereby enhancing antioxidant capacity characterized by increased levels of antioxidant enzymes (SOD, GSH, GSH‐Px) and decreased level of the lipid peroxidation marker MDA. Additionally, UA suppresses neuroinflammation, which is manifested by reduced levels of pro‐inflammatory cytokines (TNF‐α, IL‐1β, IL‐6) and elevated level of the anti‐inflammatory cytokine IL‐10. RNA transcriptome sequencing analysis further revealed that these neuroprotective effects of UA may be associated with inhibition of pathological NF‐κB activation.

**Conclusion:**

Urolithin A may exert neuroprotective effects against ischemic stroke by inhibiting oxidative stress and neuroinflammation. Collectively, this study provides theoretical support for the clinical translation of UA as a poststroke neuroprotective agent.

## Graphical abstract

Urolithin A (UA) treatment enhances the Nrf2/HO‐1‐mediated antioxidant capacity against ischemic injury postischemic stroke, while also effectively suppressing neuroinflammatory responses. Both of these regulatory effects are likely associated with the NF‐κB signaling pathway, which in turn inhibits neuronal apoptosis and contributes to the improvement of neurological function outcomes.

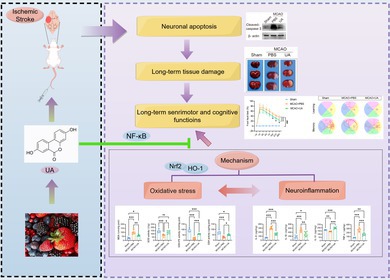

## 1 Introduction

Ischemic stroke (IS), accounting for approximately 80%–87% of all stroke cases [1, 2], is a devastating cerebrovascular disease characterized by sudden interruption of cerebral blood flow, leading to irreversible neuronal damage, severe neurological deficits, and even death [3, 4]. Its sequelae involve interconnected pathological processes: cerebral ischemia disrupts brain energy metabolism, triggering excitotoxicity, calcium overload, mitochondrial dysfunction, and excessive reactive oxygen species (ROS) production [5, 6]. ROS damage biomolecules and activate pro‐inflammatory pathways (e.g., NF‐κB, NLRP3 inflammasome), promoting pro‐inflammatory cytokine release and exacerbating neuronal injury and cognitive dysfunction [7, 8]; they also induce programmed cell death, damaging neural networks [9, 10]. Currently, IS therapies are limited, highlighting an urgent need for multi‐targeted neuroprotective strategies to improve post‐stroke functional recovery translation.

Mounting evidence has demonstrated that oxidative stress and neuroinflammation are key drivers of the initiation and cascade amplification of IS in the early phase [11, 12, 13]. During ischemia–reperfusion, a sudden influx of oxygen results in excessive ROS production, which disrupts intracellular redox homeostasis and induces lipid peroxidation, DNA damage, and mitochondrial collapse [14]. Concurrently, ROS activate inflammation‐related pathways (e.g., NF‐κB, NLRP3), triggering the release of pro‐inflammatory cytokines and further amplifying neuronal damage [12]. Moreover, IS is accompanied by the activation of programmed cell death, with apoptosis being particularly prominent in the early stages of injury. The apoptotic pathway is primarily mediated by the mitochondrial pathway, and the imbalance of molecules such as Caspase‐3, cytochrome c (Cyt‐c), and the Bax/Bcl‐2 ratio serves as a critical indicator of irreversible neuronal damage [15].

Against this pathological backdrop, the nuclear factor erythroid 2‐related factor 2 (Nrf2)/heme oxygenase‐1 (HO‐1) signaling pathway is recognized as a pivotal endogenous defense mechanism against oxidative stress and inflammatory responses [16]. Nrf2 is a core transcription factor that regulates the expression of antioxidant genes. Under oxidative stress conditions, Nrf2 dissociates from its inhibitor Kelch‐like ECH‐associated protein 1 (Keap1) and translocates into the nucleus, where it binds to antioxidant response elements (AREs) to activate the expression of a suite of antioxidant enzymes, including HO‐1, superoxide dismutase (SOD), and glutathione peroxidase (GSH‐Px) [17, 18, 19]. This activation effectively mitigates oxidative damage and attenuates inflammatory responses. Furthermore, studies have shown that the Nrf2/HO‐1 pathway can negatively regulate NF‐κB signaling activity, inhibit the release of pro‐inflammatory factors, and partially interfere with apoptotic pathways, thereby exhibiting broad neuroprotective potential [20, 21].

Urolithin A (UA) is a natural metabolite produced by gut microbiota through the biotransformation of ellagitannin‐rich polyphenols (e.g., those in pomegranates) [22]. In recent years, UA has been shown to exert robust neuroprotective effects in models of neurodegenerative diseases, such as Alzheimer's disease and Parkinson's disease [23, 24, 25]. Notably, UA possesses favorable oral bioavailability and can cross the blood–brain barrier (BBB) two critical properties for central nervous system (CNS) therapeutics [26].

Despite UA's known neuroprotection in neurodegenerative diseases, its mechanism and efficacy in IS remain unclear. Using a rat MCAO model, we found UA improved sensorimotor function and spatial learning memory. RNA‐seq showed UA‐regulated genes enriched in antioxidant/anti‐inflammatory pathways linked to Nrf2/HO‐1. In the ischemic penumbra, UA reduced neuronal apoptosis and damage. Mechanistically, UA upregulated Nrf2/HO‐1, enhanced antioxidant enzyme activity, decreased MDA, and regulated pro/anti‐inflammatory cytokines. This study is the first to confirm UA's efficacy in ameliorating post‐IS deficits and clarify its mechanism, supporting clinical translation as a post‐stroke neuroprotective agent.

## 2 Materials and Methods

### 2.1 Animal Model of IS and UA Administration

Adult male Sprague–Dawley (SD) rats (6–8 weeks, 220–250 g) were obtained from Xingkang Shengwu Animal Center, housed with free access to food and water. All experimental procedures were approved by the Animal Care and Use Committee of Shandong First Medical University and complied with NIH laboratory animal care guidelines. Efforts were made to minimize animal usage and suffering.

IS models were established by MCAO as described [27]. Briefly, isoflurane‐anesthetized rats were supine‐fixed; neck disinfection and midline incision exposed the right CCA, ICA and ECA. CCA was slipknotted, distal ECA permanently ligated and proximal ECA loosely ligated. Distal ICA was clamped, a nylon filament inserted via ECA to occlude the right MCA, followed by tying proximal ECA and releasing CCA slipknot. After 2 h occlusion, filament withdrawal induced reperfusion, with tail vein drug delivery at 1 h post‐reperfusion. Sham rats underwent identical surgery without MCAO. All procedures were performed by investigators blinded to grouping.

Urolithin A was dissolved in DMSO to 20 mg/mL stock solution, then diluted 100‐fold with PBS for use. UA (3 mg/kg) was administered via tail vein injection 1 h post‐surgery.

Before surgery, rats were randomly assigned to the Sham, MCAO+PBS, and MCAO+UA groups using a random number method. Group allocation and treatment codes were concealed from investigators performing behavioral tests, tissue processing, histological quantification, biochemical assays, and data analysis. Behavioral assessments were conducted in a blinded manner. For TTC staining, immunofluorescence, Western blotting, and ELISA, samples and images were coded, and group identities were revealed only after quantitative analyses were completed.

### 2.2 Behavioral Assessments

This study assessed sensorimotor dysfunctions in rats using the Garcia Score, Adhesive‐Removal Test, Foot‐fault Test and Rotarod Test on 1, 3, 5, 7, 14, 21, and 28 days post‐IS. The Morris Water Maze test (MWM) was used to evaluate spatial learning. The specific methods were referred to previous studies [28].

### 2.3 Immunofluorescence Staining

Immunofluorescence staining was performed as previously described [2]. In brief, rats were transcardially perfused with cold sterile PBS (pH 7.4) followed by 4% PFA; brains were postfixed in 4% PFA at 4°C overnight, dehydrated in sequential 20%/30% sucrose (4°C until sinking), embedded in OCT compound (Sakura Finetek, Japan), and sectioned into 20 μm‐thick coronal slices via a cryostat (Leica CM1950, Germany). Selected slices were permeabilized with 0.3% Triton X‐100/PBS for 30 min at RT, washed with PBS (3 times, 5 min/time), and blocked in 5% donkey serum/PBS for 1 h at RT to reduce nonspecific binding. Tissues were then incubated with primary antibodies at 4°C overnight: Anti‐NeuN (Wuhan Sanying, 1:200), Anti‐Cleaved Caspase‐3 (Wuhan Sanying, 1:200), and Anti‐Nrf2 (Abcam, 1:200). After PBS washes, slices were incubated with Goat Anti‐Rabbit (Abcam, 1:1000), Goat Anti‐Mouse (Abcam, 1:1000) for 1 h at RT in the dark, washed with PBS, stained with DAPI (Sigma‐Aldrich, USA; 1:1000 in PBS) for 5 min at RT, and mounted with anti‐fade medium (Thermo Fisher, USA) post‐final PBS wash. Fluorescent images were acquired via an FV1200 confocal microscope (Olympus, Japan) with consistent excitation/emission parameters; quantification was done via ImageJ software.

### 2.4 HE Staining

Selected brain sections were mounted on adhesion microscope slides, air‐dried, and rinsed with a graded ethanol series (100%, 95%, 80%, and 75% in distilled water). After washing, tissues were stained with hematoxylin (2 g/L) for 2 min, followed by distilled water rinsing. Sections were then immersed in hydrochloric acid/ethanol solution for 30 s, soaked in distilled water for 15 min, dipped in 1% eosin solution for 10 s, and rinsed with distilled water. Finally, dehydration was performed with anhydrous ethanol, tissues were mounted with neutral resin, and images were captured using a light microscope.

### 2.5 2,3,5‐Triphenyltetrazolium Chloride (TTC) Staining

At 72 h after reperfusion, experimental animals were anesthetized and decapitated. Brains were immediately harvested to measure infarct volume: sequentially sectioned into six 2‐mm‐thick coronal slices, which were incubated in 2% TTC solution at 37°C for 30 min. After staining, slices were photographed, and infarct volume was quantified using the ImageJ software. Infarct volume percentage was calculated as (infarct volume/total brain volume) × 100%.

### 2.6 Western Blotting

Western blot was performed as previously described [2]. Brain tissue samples (100 mg, including infarcted and peri‐infarct zones) were placed into EP tubes containing 1 mL pre‐cooled lysis buffer and homogenized thoroughly. The homogenates were centrifuged at 12,000 g for 5 min at 4°C, and supernatants were transferred to pre‐cooled centrifuge tubes. Total protein concentration was determined using a BCA protein assay kit. For protein denaturation, sample loading buffer was added at a 1:4 ratio, and samples were boiled at 95°C for 5 min. Proteins were separated by SDS‐PAGE and transferred onto PVDF membranes. Membranes were washed three times with Tris‐buffered saline containing 0.1% Tween‐20 (TBST) for 10 min each, then blocked with 5% non‐fat milk in TBST for 2 h at room temperature. After three additional TBST washes (10 min each), membranes were incubated overnight at 4°C with primary antibodies: anti‐Cleaved Caspase‐3 (Wuhan Sanying, 1:200), anti‐Nrf2 (Abcam, 1:200), and anti‐HO‐1 (Cell Signaling Technology, 1:200), β‐actin (Cell Signaling Technology, 1:200). The following day, membranes were washed three times with TBST (10 min each) and incubated with Goat Anti‐Rabbit IgG H&L (HRP) (Abcam, 1:1000), Goat Anti‐Mouse IgG H&L (HRP) (Abcam, 1:1000) for 1.5 h at room temperature. After three final TBST washes, protein bands were visualized using ECL chemiluminescence reagents (Thermo Fisher Scientific, USA). Each experiment was performed in triplicate, and band intensities were quantified using the ImageJ software.

### 2.7 Enzyme‐Linked Immunosorbent Assay (ELISA)

Levels of IL‐6, IL‐10, IL‐1β, and TNF‐α in rat brain tissues were measured using commercial ELISA kits (Solarbio, Beijing, China), while superoxide dismutase (SOD), malondialdehyde (MDA), glutathione (GSH), and glutathione peroxidase (GSH‐Px) levels were determined with kits from Biyuntian (Shanghai, China), following the manufacturers' protocols. All samples were assayed in triplicate. The optical density (OD) of each well was measured at 450 nm using a microplate reader, and concentrations of the inflammatory cytokines and oxidative stress markers were calculated based on their respective standard curves.

### 2.8 RNA Sequencing and Analysis

Three sets of brain tissues were rapidly dissected on ice, immersed in 5× volume RNAlater solution, incubated at 4°C overnight, and stored at −80°C. RNA sequencing and initial data processing were performed by Heyuan Bio (Beijing, China). Raw read counts were normalized with DESeq2; PCA, differential expression analysis, and KEGG pathway enrichment for DEGs were conducted. Heatmaps and other plots visualized DEGs and enriched pathways.

### 2.9 Statistical Analysis

Statistical analyses were performed using the SPSS Statistics 20 and the GraphPad Prism software. All quantitative data are presented as the mean ± standard error of the mean (mean ± SEM). The Shapiro–Wilk test was used to assess normality: for normally distributed data, one‐way or two‐way ANOVA was applied to compare intergroup differences, followed by Bonferroni post hoc tests for multiple comparisons; for non‐normally distributed data, the Kruskal‐Wallis test was used. A two‐tailed *p* value < 0.05 was considered statistically significant.

## 3 Results

### 3.1 Urolithin A Improves Long‐Term Neurological Function After Ischemic Stroke

**FIGURE 1:**
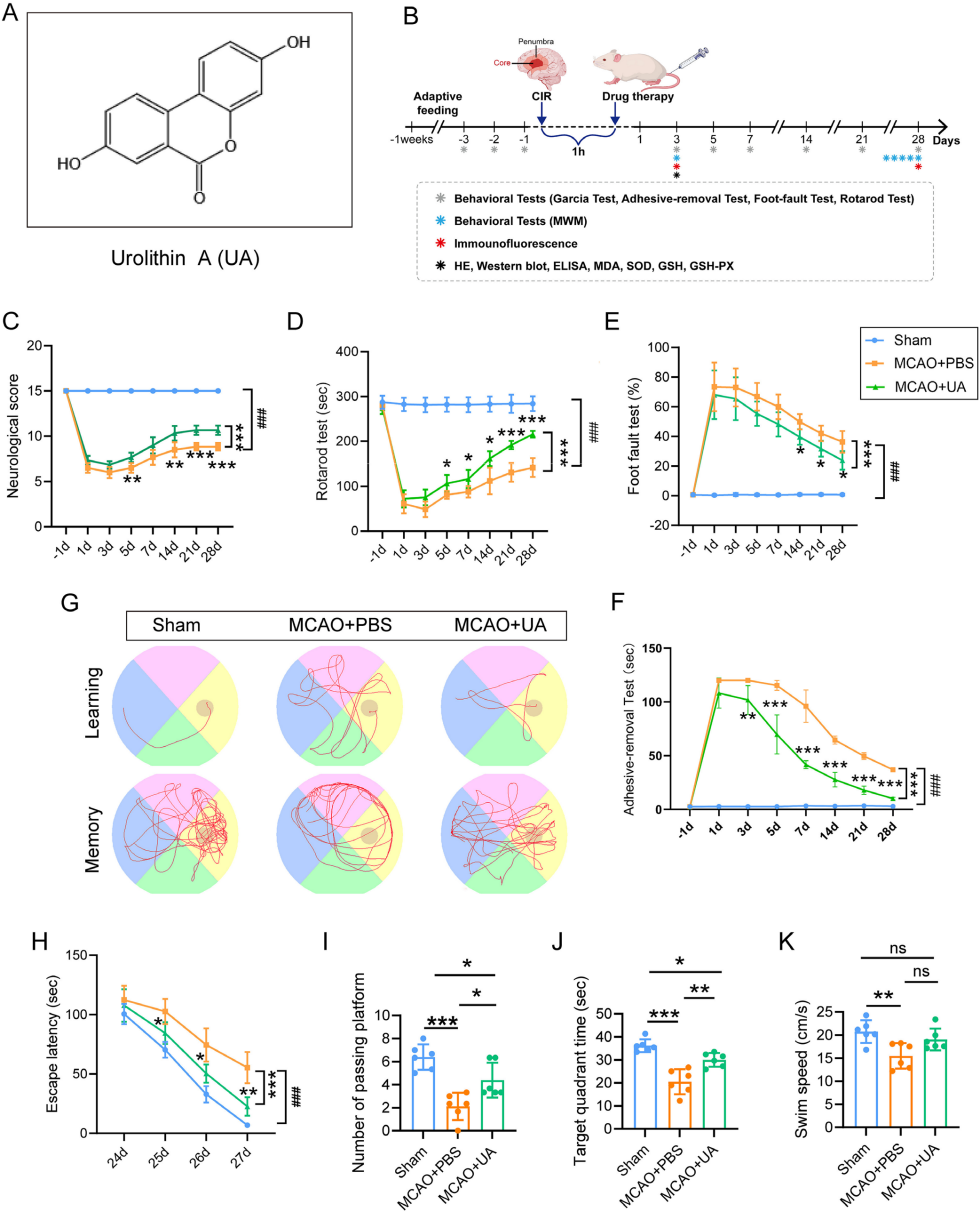
UA treatment improves long‐term sensorimotor and cognitive functions following IS. (A) Molecular formula of Urolithin A (UA). (B) Experimental timeline. (C–F) Sensorimotor function assessments post‐MCAO/Sham: Neurological function scores (C), rotarod test (D), foot‐fault test (E), adhesive removal test (F). (G) Representative swimming trajectories of the WMW test. (H) Escape latency during acquisition training. (I‐K) MWM probe trial: Platform crossings (I), target quadrant time (J), swimming speed (K). Data are presented as means ± SEM. *p < 0.05, **p < 0.01, ***p < 0.001, ^###^ *p* < 0.001, ns: Not significant, as indicated.

Urolithin A is a benzocoumarin compound, specifically a derivative of dibenzo‐α‐pyran‐6‐one substituted with two hydroxyl groups (Figure 1A). To evaluate the long‐term effects of UA on neurological function after IS, a 28‐day behavioral study was performed using neurological function scoring, rotarod, adhesive removal, foot‐fault tests, and MWM (Figure 1B). Neurological function scores showed no deficits in the Sham group, severe impairments in the MCAO+PBS group, while UA treatment significantly alleviated IS‐induced neurological deficits (Figure 1C). Rotarod test (motor coordination) revealed shorter latency in MCAO+PBS rats; UA treatment significantly improved rotarod performance from post‐stroke Day 5 (Figure 1D). Sensorimotor function tests showed increased foot‐fault rate and prolonged adhesive‐removal time in MCAO+PBS rats. UA significantly reduced adhesive detection time from Day 3 (Figure 1F) and improved foot‐fault performance from Day 14 (Figure 1E), indicating enhanced sensorimotor function.

MWM test (Days 24–28) assessed spatial learning and memory (Figure 1G). During acquisition training, MCAO+PBS rats had longer escape latency than Sham rats, which was reduced by UA treatment (Figure 1H); locomotor speed showed no significant differences between MCAO subgroups (Figure 1K). In the probe trial, MCAO+PBS rats spent less time and made fewer crossings in the target quadrant, while UA significantly increased both parameters (Figure 1I,J), suggesting protection against spatial memory impairment.

Collectively, MCAO‐induced rats displayed deficits in spatial learning, memory, and motor abilities, which were effectively mitigated by UA treatment.

### 3.2 Urolithin A Reduces Acute Infarct Size and Long‐Term Tissue Damage After Ischemic Stroke

**FIGURE 2:**
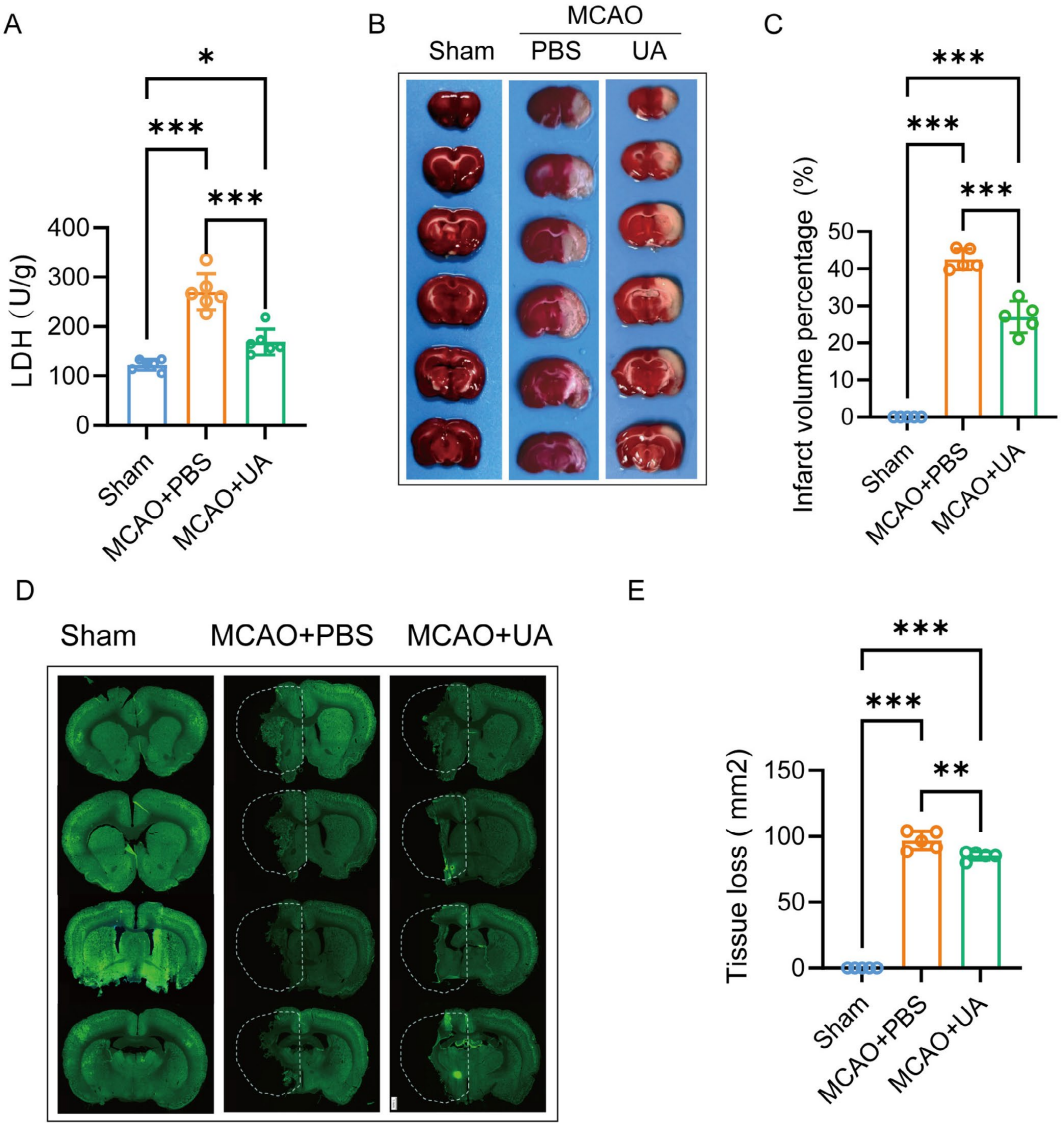
UA treatment reduces acute infarct size and long‐term tissue damage after IS. (A) LDH content (U/g) in rat brain tissue 3 days post‐MCAO. (B) Representative TTC‐stained coronal brain sections of rats 3 days post‐MCAO. (C) Quantification of infarct area percentage (%) based on TTC staining. (D‐E) Representative images of NeuN immunofluorescence staining and quantification of NeuN‐negative (loss) area. Data are presented as means ± SEM. *p < 0.05, **p < 0.01, ***p < 0.001.

To investigate UA's short‐ and long‐term effects on cerebral infarction, infarct severity was quantified via multiple assays. Lactate dehydrogenase (LDH), a sensitive marker for brain injury with release positively correlated to cellular damage [29], was measured at 3 days post‐IS. The MCAO+PBS group had the highest LDH levels, while UA treatment significantly reduced LDH release, indicating attenuated brain damage (Figure 2A).

To visually evaluate the proportion of infarct volume in brain tissue, fresh coronal brain sections were stained with TTC at 3 days after MCAO surgery. In TTC staining, viable tissue appears deep red, while the infarcted area in the ipsilateral cerebral hemisphere presents as white (Figure 2B). The TTC staining results revealed that brain slices from the Sham group were uniformly red, with no signs of ischemic necrosis. In contrast, the MCAO+PBS group exhibited a large white region in the ipsilateral hemisphere, which corresponded to the territory of the middle cerebral artery, indicating extensive ischemic necrosis. The volume of the white infarcted area in the MCAO+PBS group was significantly larger than that in the Sham group, whereas the UA‐treated group showed a marked reduction in the white infarcted area (Figure 2C). These findings suggest that UA treatment reduces the short‐term infarct volume in rats after IS. To further explore the effect of UA on long‐term tissue damage, NeuN staining was conducted on brain sections from rats at 28 days post‐MCAO. Quantitative analysis of the damaged area in the ipsilateral cerebral hemisphere demonstrated that the MCAO+PBS group had the largest infarct volume. In comparison, the MCAO+UA group exhibited a smaller infarct volume than the MCAO+PBS group, which was consistent with the aforementioned findings (Figure 2D,E).

In summary, urolithin A not only reduces the short‐term infarct volume but also exerts protective effects against long‐term tissue damage induced by IS.

### 3.3 Urolithin A Treatment Inhibits Neuronal Apoptosis After Ischemic Stroke

Irreversible neuronal death occurs in the infarct core, whereas the surrounding ischemic penumbra—characterized by partially preserved perfusion and reversible structural damage—contains neurons highly sensitive to ischemia–reperfusion injury [30]. We hypothesized that UA protects the ischemic penumbra to reduce infarct volume. Using morphological and molecular approaches, we evaluated UA's effects on neuronal apoptosis in the cortical penumbra of a cerebral ischemia–reperfusion model.

**FIGURE 3:**
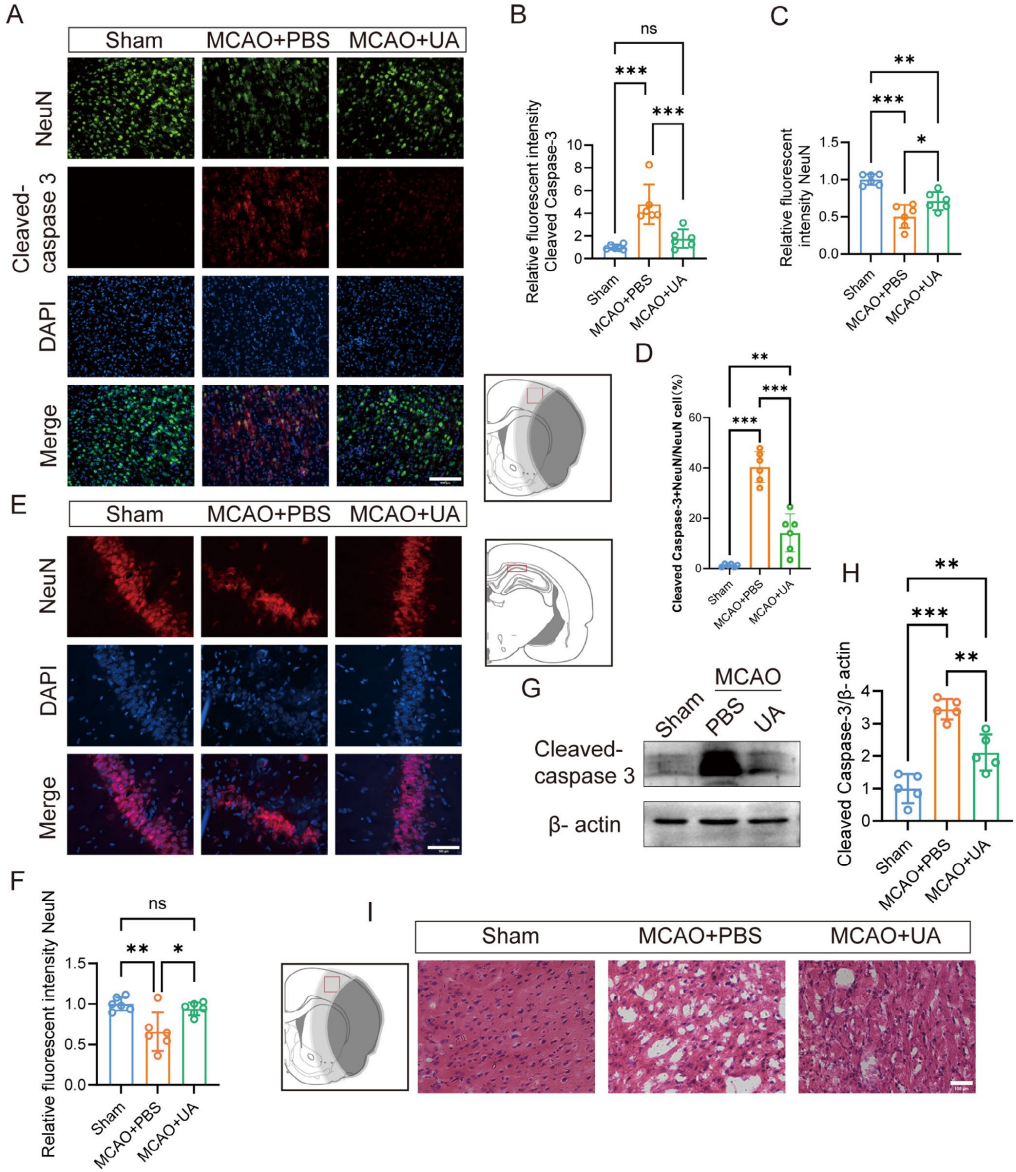
UA treatment reduces neuronal apoptosis in the cerebral cortex and hippocampus after IS. (A) Representative NeuN/Cleaved Caspase‐3 co‐staining images in ischemic penumbra 3 days post‐MCAO. Scale bar: 200 μm. (B, C) Quantitative analysis of NeuN and Cleaved Caspase‐3 mean fluorescence intensity. (D) Ratio of Cleaved Caspase‐3/NeuN double‐positive to total NeuN‐positive cells. (E‐F) Representative NeuN immunofluorescence images and quantification of NeuN fluorescence intensity in CA1. Scale bar: 100 μm. (G, H) Representative western blots of Cleaved Caspase‐3 and quantification of its relative expression. (I) Representative HE staining of cortical ischemic penumbra. Scale bar: 100 μm. Schematic shows image‐captured regions (A, E, I). Data are presented as means ± SEM. *p < 0.05, **p < 0.01, ***p < 0.001, ns: Not significant, as indicated in the figures.

Immunofluorescence staining for NeuN (viable neurons) and Cleaved Caspase‐3 (apoptotic neurons) showed abundant NeuN‐positive cells with minimal Cleaved Caspase‐3 signals in Sham penumbra (Figure 3A). MCAO+PBS rats exhibited reduced NeuN positivity, increased Cleaved Caspase‐3 colocalization with NeuN, and marked neuronal apoptosis. UA treatment significantly increased NeuN‐positive neurons, decreased Cleaved Caspase‐3 signals, and reduced colocalization (Figure 3B–D), confirming apoptosis inhibition. Western blot analysis showed MCAO+PBS rats had elevated Cleaved Caspase‐3 expression (*p* < 0.001 vs. Sham), which UA significantly downregulated (*p* < 0.01 vs. MCAO+PBS) (Figure 3G,H). HE staining revealed organized, intact neurons in Sham penumbra, versus disorganized cells with vacuolization and nuclear deformation in MCAO+PBS rats. UA treatment ameliorated neuronal disarray and reduced vacuolar damage (Figure 3I), visually confirming anti‐apoptotic effects. In the hippocampal CA1 region‐critical for cognition and vulnerable to I/R injury [31, 32]‐NeuN staining showed abundant, intact neurons in Sham rats, versus reduced NeuN positivity, disorganized architecture, and vacuolar damage in MCAO+PBS rats. UA treatment increased NeuN‐positive neurons, improved cellular organization, and mitigated ischemic injury (Figure 3E,F).

In summary, UA inhibits neuronal apoptosis in the cortical ischemic penumbra via reduced Cleaved Caspase‐3 expression and enhanced NeuN positivity, while protecting hippocampal CA1 neurons to exert neuroprotective effects against ischemic brain injury.

### 3.4 Urolithin A Treatment Alters the Cerebral Transcriptome and Inhibits Apoptosis‐Related Gene Pathways After IS

To investigate the molecular mechanisms of UA's neuroprotection after IS, RNA sequencing and bioinformatics analyses were performed on infarcted brain tissue (3 days post‐surgery).

**FIGURE 4:**
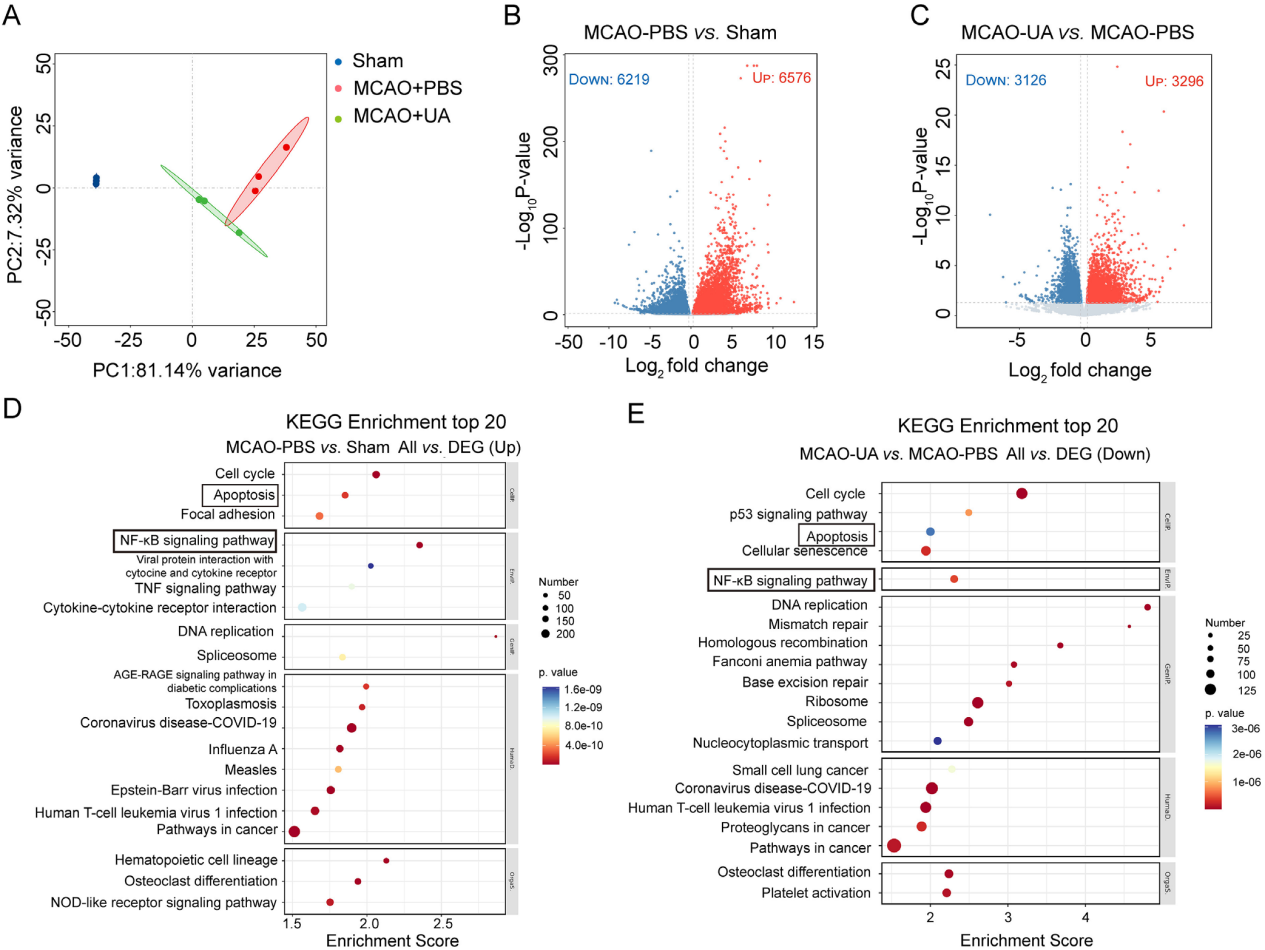
Transcriptomic differences between UA and PBS groups demonstrate inhibition of apoptosis‐related gene expression. (A) Principal Component Analysis (PCA) plot showing RNA sequencing data from ischemia–reperfusion brain tissues of three groups of rats. (B, C) Volcano plots showing upregulated and downregulated differentially expressed genes among the three groups. (D‐E) Top 20 enriched KEGG pathways: (D) Top 20 upregulated pathways in MCAO+PBS vs. Sham; (E) Top 20 downregulated pathways in MCAO+UA vs. MCAO+PBS. *n* = 3/group; criteria for panels B–E: Adjusted fold change (FC) > 1.2, |log2FC| > 0.26, and *p*‐value < 0.05.

Principal component analysis (PCA) showed clear intergroup separation, with the MCAO+UA group clustering distinctly from MCAO+PBS, indicating UA‐altered transcriptomic profiles (Figure 4A). Volcano plots (FC > 1.2, |log_2_FC| > 0.26, *p* < 0.05) revealed 6219 downregulated/6576 upregulated genes in MCAO+PBS vs. Sham, and 3126 downregulated/3296 upregulated genes in MCAO+UA vs. MCAO+PBS (Figure 4B,C).

KEGG enrichment of DEGs showed UA‐regulated genes were significantly enriched in “Apoptosis” and “NF‐κB signaling pathway” (Figure 4D,E). Importantly, UA treatment markedly suppressed the enrichment of the NF‐κB pathway. NF‐κB is a well‐established redox‐sensitive transcription factor that can be activated by excessive reactive oxygen species (ROS). Upon activation, NF‐κB translocates to the nucleus and promotes transcription of multiple pro‐inflammatory cytokines, including TNF‐α, IL‐1β, and IL‐6, thereby amplifying neuroinflammatory responses [33, 34]. Therefore, NF‐κB may function as a critical molecular hub linking oxidative stress to inflammation in the context of ischemic stroke. The transcriptomic data suggest that UA‐mediated neuroprotection may involve coordinated suppression of oxidative stress and NF‐κB–driven inflammatory cascades.

### 3.5 UA May Exert Neuroprotective Effects by Inhibiting Oxidative Stress, Potentially via the Nrf2/HO‐1 Pathway

**FIGURE 5:**
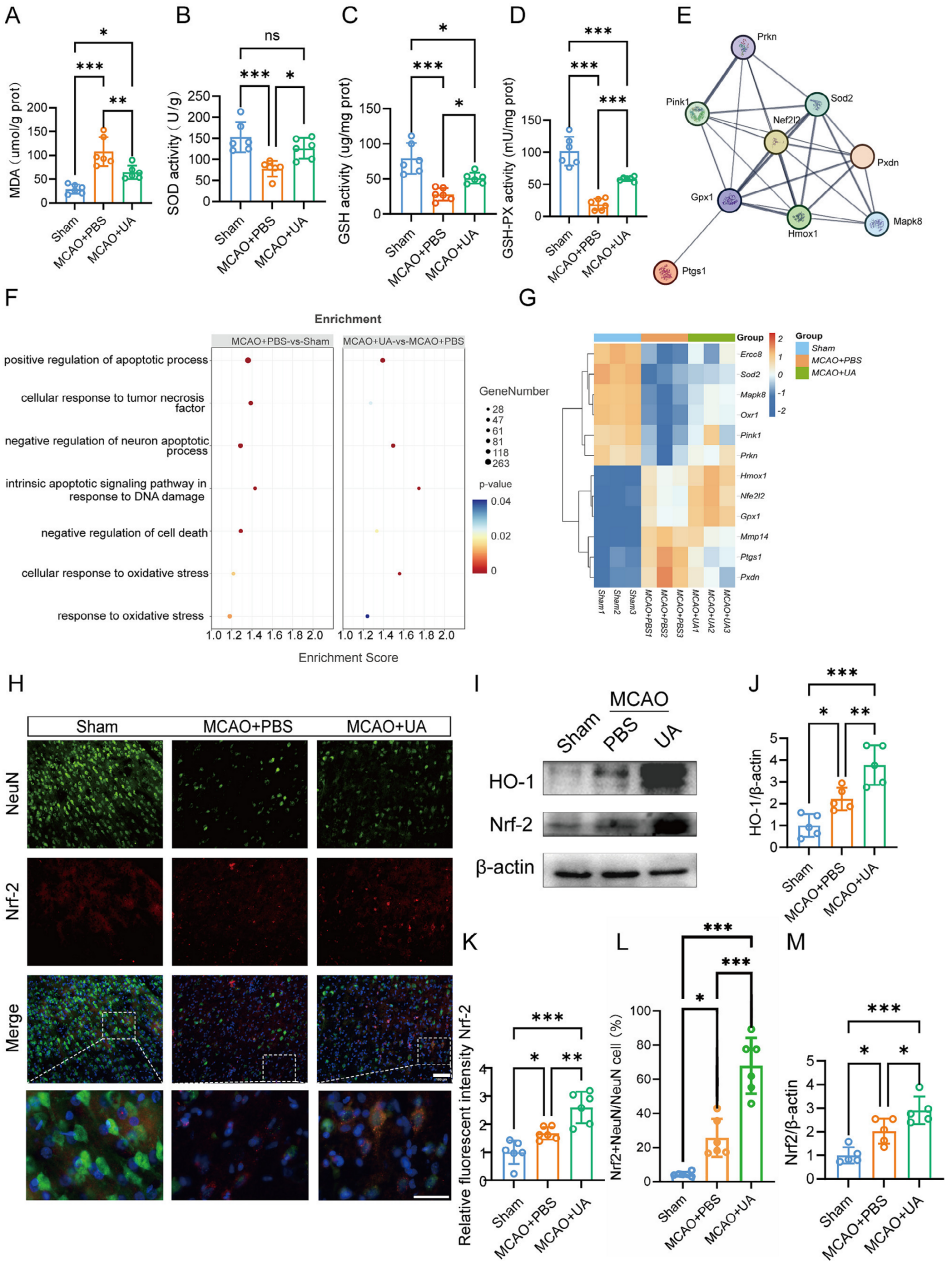
UA may inhibit oxidative stress and inflammatory responses post‐IS, potentially via the Nrf2/HO‐1 signaling pathway. (A–D) Quantification of oxidative stress markers in the ipsilateral infarcted hemisphere at 3 days post‐MCAO: (A) MDA content; (B) SOD activity; (C) GSH level; (D) GSH‐PX activity. (E) PPI network of oxidative stress‐related genes. (F) GO enrichment analysis of oxidative stress and apoptosis‐related pathways. (G) Heatmap of oxidative stress‐related signaling molecules and their interacting genes. (H) Representative NeuN/Nrf2 co‐immunofluorescence staining in the cortical ischemic penumbra at 3 days post‐MCAO. Scale bar: 100 μm (upper) and 50 μm (lower). (I) Representative western blots of Nrf2 and HO‐1 from ischemic hemispheres. (J) Quantification of relative HO‐1 protein expression. (K) Quantitative analysis of Nrf2 mean fluorescence intensity. (L) Percentage of NeuN/Nrf2 double‐positive cells relative to total NeuN‐positive cells. (M) Quantification of relative Nrf2 protein expression. Data in E‐G: *N* = 3; criteria for E, F, G: FC > 1.2, |log2FC| > 0.26, and *p* < 0.05. Data are presented as means ± SEM. *p < 0.05, **p < 0.01, ***p < 0.001.

Previous studies have shown that IS activates oxidative stress and triggers central nervous system inflammation. To determine whether UA alleviates oxidative stress following ischemic stroke, we first assessed oxidative stress‐related biochemical markers in brain tissue. MCAO+PBS rats exhibited significantly increased lipid peroxidation, as reflected by elevated MDA levels, whereas UA treatment markedly reduced MDA accumulation (Figure 5A). In parallel, antioxidant defense systems were impaired after ischemia, with decreased SOD activity and reduced GSH and GSH‐Px levels; these changes were significantly restored by UA administration (Figure 5B–D), indicating that UA effectively mitigates oxidative damage.

Subsequently, we analyzed oxidative stress‐ and apoptosis‐related pathways via Gene Ontology (GO) enrichment (Figure 5F). Compared with the MCAO+PBS group, the MCAO+UA group downregulated pathways like “positive regulation of apoptotic process” while activating “cellular response to oxidative stress”‐suggesting UA acts via coordinated inhibition of these processes. Based on differentially expressed genes (DEGs), heatmap analysis of oxidative stress‐related genes showed the UA group significantly upregulated *Nfe2l2* (encoding Nrf2), *Hmox1* (encoding HO‐1), *Sod2*, and *Gpx1*, with a gene pattern distinct from the PBS group (Figure 5G). Protein–protein interaction (PPI) network analysis revealed Nrf2 as a central node, forming a tight network with HO‐1, SOD2, and GPX1 (Figure 5E)‐indicating the Nrf2/HO‐1 pathway mediates UA's antioxidant effects.

To verify this pathway, double immunofluorescence staining of NeuN and Nrf2 was performed in the cortical ischemic penumbra at 3 days post‐MCAO (Figure 5H). NeuN staining showed the UA group had more orderly, numerous, and uniformly distributed neurons vs. the MCAO+PBS (Figure 5H). Additionally, the UA group had more NeuN/Nrf2 double‐positive cells and enhanced nuclear Nrf2 localization (Figure 5K,L), suggesting UA promotes Nrf2 nuclear translocation in viable penumbra neurons to activate antioxidant defenses. Western blot analysis validated these results: the MCAO+UA group had higher Nrf2 and HO‐1 protein levels in brain tissue vs. MCAO+PBS (Figure 5I,J,M), confirming UA activates the Nrf2/HO‐1 axis to boost antioxidant capacity and alleviate IS‐induced oxidative damage.

In summary, UA mitigates post‐IS oxidative stress by upregulating Nrf2/HO‐1 pathway molecules and enhancing antioxidant gene network activity, confirming its neuroprotective effects centered on this pathway.

### 3.6 Urolithin A Suppresses NF‐κB–Mediated Neuroinflammation After Ischemic Stroke

We previously found UA may regulate neuronal apoptosis via NF‐κB (Figure 4) and oxidative stress via Nrf2/HO‐1 (Figure 5). Given the central involvement of NF‐κB in ischemic stroke (IS)–triggered inflammation, we explored whether UA modulates post‐stroke inflammation via NF‐κB‐dependent signaling.

**FIGURE 6:**
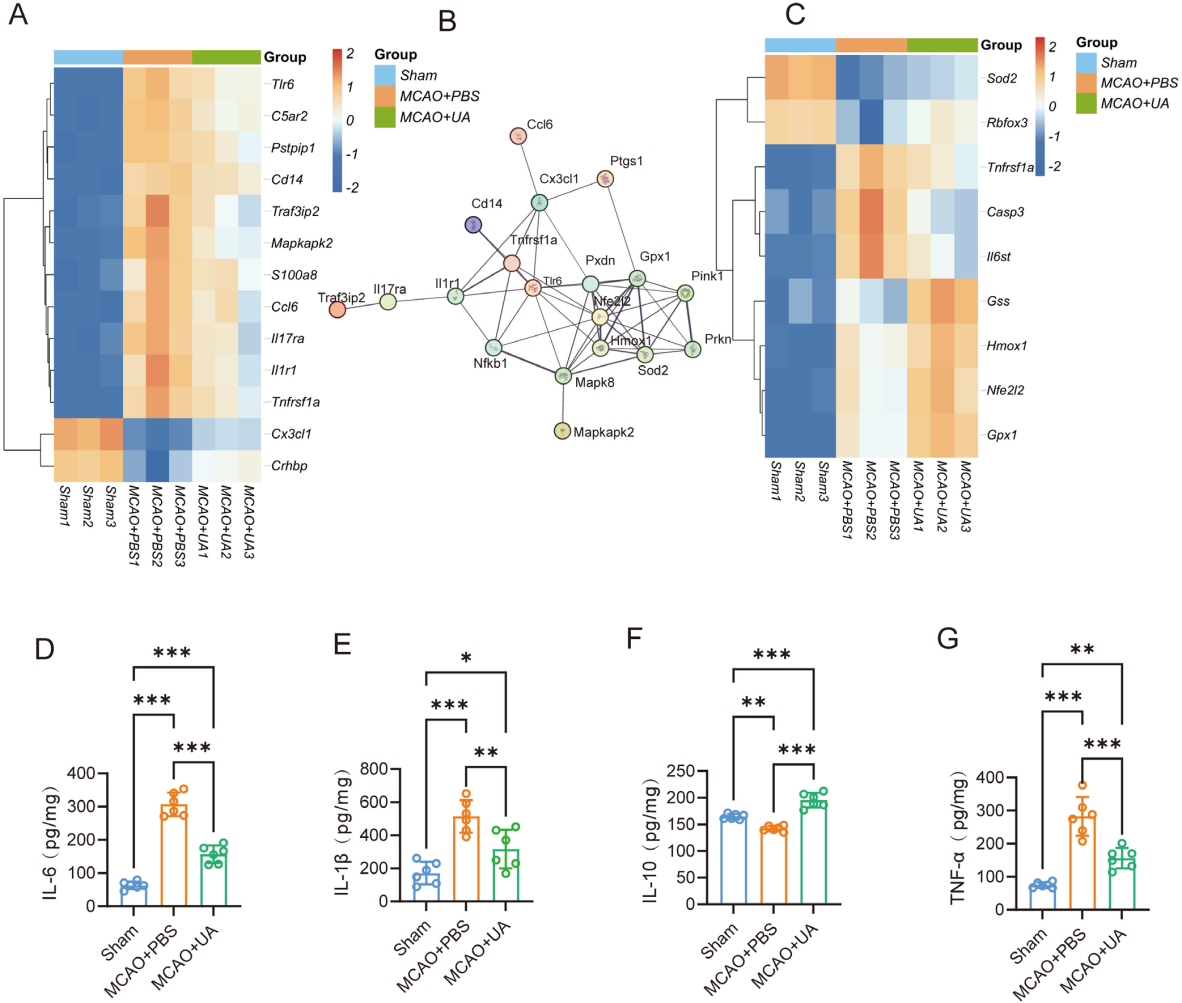
Urolithin A suppresses NF‐κB–mediated neuroinflammation after ischemic stroke. (A) Heatmap of relative expression levels of inflammation‐related signaling molecules and their interacting genes. (B) Protein–protein interaction (PPI) network of inflammation‐related genes. (C) Heatmap of differential expression levels of genes associated with oxidative stress, inflammation, and apoptosis. (D–G) ELISA quantification of cytokine concentrations in the ipsilateral infarcted hemisphere at 3 days post‐MCAO: (D) IL‐6; (E) IL‐1β; (F) IL‐10; (G) TNF‐α. Data are presented as means ± SEM. *p < 0.05, **p < 0.01, ***p < 0.001, ns: Not significant (as indicated).

Transcriptomic analysis showed MCAO+PBS rats had upregulated pro‐inflammatory genes (*C5ar2, Tnfrsf1a*, etc.) and downregulated antioxidant genes; UA reversed these changes and upregulated anti‐inflammatory genes (e.g., *Cx3cl1*, Figure 6A). PPI network identified Nfkb1 as a hub connecting pro‐inflammatory and oxidative stress‐related genes (Figure 6B). A candidate gene heatmap confirmed UA restored antioxidant genes (*Nfe2l2, Hmox1*, etc.), suppressed pro‐inflammatory/apoptotic genes (Figure 6C). ELISA quantification revealed that ischemic injury significantly elevated pro‐inflammatory cytokines IL‐6, IL‐1β, and TNF‐α, whereas UA treatment markedly reduced their levels (Figure 6D,E,G). Conversely, the anti‐inflammatory cytokine IL‐10 was significantly increased following UA administration (Figure 6C). These findings indicate that UA attenuates neuroinflammation after ischemic stroke, potentially through inhibition of NF‐κB signaling.

Collectively, UA exerts neuroprotection by: (1) activating Nrf2/HO‐1, reducing MDA, and restoring antioxidant defenses; (2) suppressing neuroinflammation via regulating cytokines, supporting its potential as a multi‐target post‐stroke agent.

## 4 Discussion

This study is the first to systematically evaluate the neuroprotective effects of UA in a MCAO model. Results demonstrate that UA significantly improves cognitive and neurobehavioral deficits following ischemic stroke. Furthermore, we elucidate that UA exerts its potential neuroprotective effects primarily via the Nrf2/HO‐1 signaling pathway, acting through multiple mechanistic layers. These findings provide experimental evidence supporting the translational application of UA in the intervention of ischemic brain injury.

Ischemic stroke involves highly complex pathological processes, with core mechanisms including oxidative stress, mitochondrial dysfunction, excitotoxicity, inflammatory responses, and various forms of programmed cell death [35, 36]. Although reperfusion post‐ischemic stroke restores cerebral blood flow, it also exacerbates free radical release, Ca^2+^ overload, and BBB disruption. This process can induce neuronal death, axonal injury, and synaptic structural damage in critical cognitive centers, ultimately leading to secondary neuronal damage, failed neural circuit remodeling, and long‐term cognitive dysfunction [37, 38, 39, 40]. The hippocampus, a key structure for learning and memory formation, is highly susceptible to ischemia–reperfusion injury—this susceptibility directly contributes to multifaceted cognitive impairments, including deficits in spatial memory, episodic memory, attention, and executive function [41]. In the present study, neurological scoring, rotarod test, adhesive removal test, foot‐fault test, and Morris water maze experiments confirmed that rats with IS exhibited significant neurological deficits in motor coordination, sensorimotor function, spatial learning, and memory. Notably, UA treatment markedly mitigated these functional impairments.

The infarct area can be divided into the core infarct zone and the ischemic penumbra. Neurons in the core infarct zone undergo irreversible death within minutes, whereas the ischemic penumbra maintains intermediate blood flow. Although energy supply in this region is insufficient to sustain normal function, partial membrane integrity and metabolic activity are preserved—cells here remain in a “salvageable” state with potential for recovery [40]. UA reduces neuronal apoptosis in the ischemic penumbra and alleviates long‐term brain damage: At 3 days post‐MCAO, NeuN/Cleaved Caspase‐3 double immunofluorescence in the cortical ischemic penumbra showed decreased Cleaved Caspase‐3 expression and fewer double‐positive cells in MCAO+UA rats; Western blot confirmed reduced Cleaved Caspase‐3 protein levels. NeuN staining at 28 days post‐MCAO further verified reduced infarct volume, confirming UA's long‐term neuroprotection.

In ischemic stroke, oxidative stress and neuroinflammation are key pathological processes that trigger neuronal death and functional impairment [42, 43]. Excessive accumulation of ROS not only impairs mitochondrial membrane potential and energy metabolism but also amplifies cellular injury by activating inflammation‐related signaling pathways (e.g., NF‐κB, NLRP3) [44, 45]. The balance of redox homeostasis in vivo depends on the coordinated regulation of multiple antioxidant systems. Among these, SOD, reduced GSH, GSH‐Px, and MDA are widely used as critical biomarkers for assessing oxidative stress levels [16]. Therefore, identifying intervention strategies with dual antioxidant and anti‐inflammatory properties is a key focus of current stroke treatment research.

Importantly, NF‐κB signaling serves as a pivotal molecular bridge linking oxidative stress to neuroinflammation after ischemic stroke. Accumulation of ROS during ischemia–reperfusion injury can trigger IκBα degradation and promote nuclear translocation of NF‐κB p65, thereby inducing transcription of multiple pro‐inflammatory cytokines such as TNF‐α, IL‐1β, and IL‐6. Sustained NF‐κB activation further amplifies inflammatory cascades and aggravates neuronal apoptosis [33]. In this context, inhibition of NF‐κB represents a key mechanism through which antioxidant interventions can simultaneously suppress downstream neuroinflammatory damage.

In the present study, transcriptomic enrichment analysis demonstrated that NF‐κB signaling was significantly activated following MCAO, whereas UA treatment markedly attenuated this pathological activation. Combined with the observed reduction in pro‐inflammatory cytokines, these findings suggest that UA suppresses excessive NF‐κB activation induced by ischemia–reperfusion injury, thereby mitigating downstream inflammatory damage.

Additionally, RNA‐seq analysis in this study confirmed that UA significantly downregulates apoptosis‐related pathways while activating antioxidant and anti‐inflammatory pathways. Gene interaction network analysis further revealed that Nrf2/HO‐1 occupies a central position. We thus hypothesize that UA inhibits IS‐induced oxidative stress responses by activating the Nrf2/HO‐1 signaling pathway. Nrf2 is a key transcription factor under oxidative stress conditions: under physiological states, Nrf2 binds to its inhibitory protein Keap1; however, when intracellular ROS levels increase, Nrf2 dissociates from the Keap1 complex, translocates to the nucleus, and upregulates the expression of a series of antioxidant genes (including HO‐1), thereby establishing a cellular self‐defense system [18]. UA promotes the nuclear translocation of Nrf2 and enhances HO‐1 expression, which in turn scavenges ROS, alleviates lipid peroxidation, and improves mitochondrial function. To further validate this, we quantified the activities of SOD, GSH, and GSH‐Px, as well as MDA content in brain tissues. Results showed that UA significantly reduced MDA levels and increased the activities of SOD, GSH, and GSH‐Px in the brains of MCAO rats. Consistent with previous studies, these findings indicate that UA exerts sustained neuroprotective effects by modulating the endogenous antioxidant defense system.

The Nrf2/HO‐1 pathway has been demonstrated to be crucial for protecting various neurons against inflammatory damage [46]. HO‐1 not only degrades the pro‐oxidant heme to release anti‐inflammatory products (e.g., carbon monoxide, iron ions, biliverdin) but also negatively regulates NF‐κB signaling activity, thereby reducing the expression of pro‐inflammatory cytokines [47, 48], This is consistent with our RNA‐seq enrichment results showing that NF‐κB signaling was significantly upregulated after ischemic injury and was markedly suppressed by UA treatment. These findings further support the notion that NF‐κB acts as a central hub integrating oxidative stress and inflammatory responses in ischemic stroke, and that UA exerts neuroprotection by attenuating its pathological activation. In the present study, RNA‐seq and functional analyses confirmed UA reduced pro‐inflammatory cytokine levels. We speculate UA's anti‐inflammatory effects are mediated via the Nrf2/HO‐1 axis, which remodels the immune microenvironment, improves post‐stroke conditions, and promotes neural repair and plasticity recovery.

Despite these encouraging findings, the neuroinflammatory correlation analysis in the present study remains preliminary, and further mechanistic investigations are warranted. Despite the observed reduction of inflammatory cytokines following UA treatment, it should be noted that the neuroinflammation‐related correlation analysis in the present study remains relatively preliminary. Our current evaluation was mainly based on transcriptomic profiling and ELISA quantification of representative cytokines, which reflects an overall inflammatory trend but does not fully establish a causal mechanistic relationship. In particular, although NF‐κB was identified as a key hub linking oxidative stress and inflammation, direct validation using pathway‐specific inhibitors or genetic manipulation was not performed. Moreover, cell‐type–specific inflammatory responses of microglia, astrocytes, and infiltrating immune cells were not separately characterized. Future studies employing targeted interventions and single‐cell–level analyses will be necessary to further elucidate the precise regulatory network underlying UA‐mediated neuroimmune modulation after ischemic stroke.

Collectively, UA synergistically activates the Nrf2/HO‐1 pathway to scavenge oxidative stress‐induced ROS and suppress pro‐inflammatory cytokine release in brain tissues, exhibiting dual antioxidant/anti‐inflammatory properties and potential for post‐stroke intervention [49]. Notably, as a gut microbiota‐derived polyphenol metabolite, UA has favorable oral bioavailability and BBB permeability, supporting clinical translation [50], and consistent neuroprotective effects in neurodegenerative disease models [51, 52, 53]. However, this study has limitations: first, the optimal dosing regimen and therapeutic time window of UA require further clarification; second, although the ischemic stroke model used herein closely mimics clinical pathological processes, it cannot fully replicate the complexity of human stroke. Therefore, future studies integrating clinical samples and multi‐species validation are warranted to further elucidate the therapeutic mechanisms of UA and identify its target patient populations. Finally, serum biochemical markers of hepatic and renal function were not assessed, although liver and kidney histology showed no obvious toxicity (Supplementary Figure 1). Future studies should include more comprehensive toxicological analyses to better establish the safety profile of Urolithin A.

## 5 Conclusions

In summary, UA treatment exerts multi‐targeted synergistic regulation by inhibiting the activation of the NF‐κB signaling pathway and activating the Nrf2/HO‐1 signaling pathway. This dual regulation attenuates oxidative stress and neuroinflammatory responses, thereby reducing neuronal apoptosis and conferring promising neuroprotective potential. The present study provides experimental evidence supporting the translational application of UA in ischemic stroke and offers novel insights and theoretical basis for developing natural small‐molecule compounds as therapeutic strategies for stroke intervention.

## Author Contributions

Yanxin Shao, Lina Feng and Yanchun Li contributed equally to this work. They conducted major experiments, analyzed all data, and drafted the original manuscript. Weixia Yang, Hong Shi and Leilei Mao conceived and designed the research study. Yichen Cai, Hui Yuan, Qin Tang and Mingfeng Yang completed partial experimental work. Leilei Mao revised the manuscript with critical intellectual input. All authors reviewed the manuscript and approved the final submitted version.

## Funding

This work was supported by the Natural Science Foundation of Shandong Province (ZR2022MH246 to LL.M., ZR2024MH100 to H.Y.), the China Postdoctoral Science Foundation (2024M760520 to LL.M), Shandong Province Medical Health Science and Technology Development Plan Project (202313011384 to LN. F), Tai'an Science and Technology Innovation Development Project (2024NS378 to Q.T), Project of Shanghai Science and Technology Innovation Action Plan (25SF1907705 to WX. Y).

## Ethics Statement

All animal experiments in this study were approved by the Animal Care and Use Committee of the Second Affiliated Hospital of Shandong First Medical University (approval number 2024–018) and were performed according to ARRIVE guidelines.

## Consent

The authors have nothing to report.

## Conflicts of Interest

The authors declare no conflicts of interest.

## Supporting information


**Figure S1:** Representative hematoxylin and eosin (H&E) staining of liver and kidney tissues in Sham, MCAO+PBS, and MCAO+UA groups. Liver sections are shown in the upper panel, and kidney sections are shown in the lower panel. No obvious histopathological alterations were observed in the liver and kidney tissues among the three groups, suggesting that UA exhibits a favorable safety profile. Scale bar: 200 μm.

## Data Availability

The data used to support the findings of this study are available from the corresponding author upon request.
