# Supplementary material for: Urolithin A Exerts Neuroprotective Effects Against Ischemic Stroke by Inhibiting Oxidative Stress and Neuroinflammation
Source: CNS Neurosci Ther. 2026 Aug 12;32(8):e71081. doi: 10.1002/cns.71081 (PMC13469178; doi:10.1002/cns.71081)
Supplement: Supplementary file 1 [file CNS-32-e71081-s001.docx]

**Supplementary material**

**Urolithin A exerts neuroprotective effects against ischemic stroke by inhibiting oxidative stress and neuroinflammation**

Yanxin Shao^#1^, Lina Feng^#1,2^, Yanchun Li^#1^, Yichen Cai^3^, Hui Yuan^1^, Qin Tang^1^, Mingfeng Yang^1^, Weixia Yang*^4^, Hong Shi*^2^, Leilei Mao*^1,5^

1. The Second Affiliated Hospital, Shandong First Medical University & Shandong Academy of Medical Sciences, Tai’an, Shandong Province, 271000, China
2. Department of Anesthesiology, Shanghai Pulmonary Hospital, School of Medicine, Tongji University, Shanghai, 200433, China
3. Department of Neurology, The Third Affiliated Hospital of Qiqihar Medical University, Qiqihar, Heilongjiang, 161002, China
4. Department of Neurology, Qingpu Branch of Zhongshan Hospital Affiliated to Fudan University, Shanghai, 201700, China
5. The National Clinical Research Center for Aging and Medicine, Department of Hand and Upper Extremity Surgery, Jing'an District Central Hospital, Fudan University, Shanghai, 200040, China

^#^ These authors contributed equally to this work.

*Corresponding author.

Leilei Mao

Email: leilei-0318@163.com.

Or

Weixia Yang

Email: yangweixia182@163.com

Or

Hong Shi

Email: 13651958255@139.com.

**Supplemental Figures 1and the Figure Legend**

**Figure. S1** Representative hematoxylin and eosin (H&E) staining of liver and kidney tissues in Sham, MCAO+PBS, and MCAO+UA groups. Liver sections are shown in the upper panel, and kidney sections are shown in the lower panel. No obvious histopathological alterations were observed in the liver and kidney tissues among the three groups, suggesting that UA exhibits a favorable safety profile. Scale bar: 200 μm.
